# *Hedyotis diffusa* plus *Scutellaria barbata* Suppress the Growth of Non-Small-Cell Lung Cancer via NLRP3/NF-*κ*B/MAPK Signaling Pathways

**DOI:** 10.1155/2021/6666499

**Published:** 2021-06-14

**Authors:** Ya-Xin Lv, Hao-Ran Pan, Xin-Ying Song, Qing-Qi Chang, Dan-Dan Zhang

**Affiliations:** ^1^Institute of Interdisciplinary Integrative Medicine Research, Shanghai University of Traditional Chinese Medicine, Shanghai 201203, China; ^2^Shanghai Zhangjiang Group Middle School, Shanghai 201203, China

## Abstract

*Hedyotis diffusa* (HD) plus *Scutellaria barbata* (SB) have been widely used in antitumor clinical prescribes as one of herb pairs in China. We investigated the effect of aqueous extract from *Hedyotis diffusa* plus *Scutellaria barbata* at the equal weight ratio (HDSB11) in inhibiting the growth of murine non-small-cell lung cancer cell (NSCLC) line LLC *in vivo* and *in vitro* in this study. Compared with other aqueous extracts, HDSB11 showed the lowest IC_50_ in inhibiting cell proliferation at 0.43 mg/ml. Besides, HDSB11 effectively suppressed colony formation and induced cell apoptosis. The further assessment of HDSB11 on the murine Lewis-lung-carcinoma-bearing mouse model showed it significantly inhibited tumors' bioluminescence at the dose of 30 g crude drug/kg. Mechanistically, HDSB11 attenuated the expressions of NLRP3, procaspase-1, caspase-1, PRAP, Bcl-2, and cyclin D1 and downregulated the phosphorylation levels of NF-*κ*B, ERK, JNK, and p38 MAPK. In conclusion, HDSB11 could alleviate cell proliferation and colony formation and induce apoptosis *in vitro* and tumor growth *in vivo*, partly via NF-*κ*B and MAPK signaling pathways to suppress NLRP3 expression.

## 1. Introduction

Lung cancer is one of the most common cancers worldwide, according to the latest global cancer report released by the World Health Organization [1]. Non-small-cell lung cancer (NSCLC) accounts for about 85% of lung cancers [2]. The available options for NSCLC treatments are physical surgery, chemotherapy, radiation therapy, targeted therapy, immunotherapy, and a combination of these abovementioned therapies [3, 4].

Inflammasomes initiate inflammatory responses under various danger signals. The NLRP3 inflammasome is assembled by NOD-like receptor NLRP3, the caspase recruitment domain (ASC), and procaspase-1. Pathogen-associated molecular patterns (PAMPs) or risk-associated molecular patterns (DAMPs) induce expressions of inflammasome components NLRP3, pro-IL-1*β*, and pro-IL-18. Then, NLRP3 promotes caspase-1 into its active form to produce caspase-1 (p10 or p20) as well as matures interleukin 1*β* (IL-1*β*) and IL-18 [5, 6]. Increasing evidence indicates the NLRP3 inflammasome plays a vital role in the occurrence and development of various tumors, including lung cancer, breast cancer, endometrial cancer, and melanoma [7–11].

*Hedyotis diffusa* Willd (HD) and *Scutellaria barbata* D. Don (SB) are both heat-clearing and detoxifying herbs according to Chinese Traditional Medicine (TCM) theory and always used together to treat numerous cancers. Modern pharmacological studies confirmed that HD and SB could inhibit tumor cell proliferation, enhance the body's immunity, induce tumor cell apoptosis, and reverse drug resistance [12–18]. The clinical drug database analysis showed that *Hedyotis diffusa* Willd plus *Scutellaria barbata* D. Don (HDSB) was the core treatment as the most common herb pair for breast cancer and bladder cancer [19, 20]. The previous research found that the ethyl acetate fraction from the aqueous extract of the HDSB at an equal weight ratio (EA11) showed the anti-inflammatory effect on RAW264.7 cells stimulated with lipopolysaccharide (LPS)/interferon-*γ* (IFN-*γ*) [21].

This study aims to investigate the antitumor effect of *Hedyotis diffusa* plus *Scutellaria barbata* on lung adenocarcinoma LLC and underlying mechanisms related to NLRP3.

## 2. Materials and Methods

### 2.1. Reagent

Dulbecco's Modified Eagle Medium (DMEM), RPMI-1640, Fetal Bovine Serum (FBS), and 0.25% trypsin were obtained from Gibco (California, USA). hygromycin B, MTT, crystal violet powder, and DMSO were purchased from Sigma (California, USA). The apoptosis detection kit was purchased from BD (State of New Jersey, USA). Bioluminescent substrates were purchased from Perkin Elmer (Waltham, USA); Matrigel was purchased from Corning (New York, USA). The BCA protein concentration assay kit was from Thermo (Waltham, USA). Anti-NLPR3 was from Novus (Colorado, USA, 1 : 500); anticyclin D1 and anticaspase-1 were purchased from Abcam (England, UK, 1 : 1000); anti-p–NF–*κ*B, anti-NF-*κ*B, anti-p-ERK 1/2, anti-ERK 1/2, anti-p-JNK, anti-JNK, anti-p-P38, anti-P38, PARP, Bcl-2, anti-GAPDH, and anti-*β*-actin were purchased from Cell Signal Technology (Boston, USA, 1 : 1000). The ECL chemiluminescence developer was purchased from Millipore (Massachusetts, USA). All other chemicals were of analytical grade.

### 2.2. Preparation of Herb Extracts

Dry herbs combined in different weight ratios or used alone were extracted by water at 100°C for 2 h. These water extracts were named according to the different weight ratios of these two herbs: HD (HD alone), SB (SB alone), HDSB11 (HD : SB combined in 1 : 1), HDSB12 (HD : SB combined in 1 : 2), and HDSB21 (HD : SB combined in 2 : 1). The identification and quality control of HD and SB have been described in previous studies [21].

### 2.3. Cell Culture

Lewis lung cancer cell line stably expressed luciferase (LLC-Luc) was provided by Professor Shi-Guo Zhu (Shanghai University of Traditional Chinese Medicine). LLC-Luc cells were cultured in the DMEM medium with 10% FBS in a 37°C and 5% CO_2_ condition. Hygromycin B (250 mg/L) was added to each passage during the cell culture to keep the luciferase label continuously. BEAS-2B, a human normal lung epithelial cell line, was purchased from Beyotime Biotechnology (Jiangsu, China) and cultured in the RPMI-1640 medium with 10% FBS in 37°C and 5% CO_2_ condition.

### 2.4. Cell Viability Assay

Cells were seeded into a 96-well plate with a density of 3000 cells per well and allowed to adhere overnight. HD, SB, HDSB11, HDSB12, and HDSB21 were added at 0, 0.1, 0.3, and 1 mg/mL, respectively. After 24 h, 0.5 mg/ml of MTT was added and incubated in each group for 4 h. The cell survival rate was calculated with the control group as 100%.

### 2.5. Colony Formation Assay

Three hundred cells per well were seeded and allowed to attach in a 6-well plate overnight. Cells were treated with HDSB11 (0.5, 1 mg/ml) in serum-free DMEM medium for 24 h and replaced with 10% FBS DMEM for additional 5 days. These colonies were fixed, stained, and counted. The colony formation rate was calculated using the control group as 100%.

### 2.6. Flow Cytometry

The FITC Annexin V Apoptosis Detection Kit was used to determine the effect of HDSB11 on cell apoptosis according to the manufactures' protocol. Cells were tested using the FACSCalibur (BD Biosciences, California, USA). The proportion of apoptotic cells was calculated using ModFit LT 3.0 software (BD Biosciences, California, USA).

### 2.7. Animal Study

The male C57BL/6 mice were purchased from the Academia Sinica (Shanghai, China). 5 × 10^5^ cells suspended in matrigel were injected into the left lung. Six days after rejection, mice were randomly divided into two groups according to bioluminescence density. The mice in the HDSB11 group were orally administrated with HDSB11 (4.7 g/kg/d, equal to 30 g crude drug of clinical dose), and the mice in the model group were administrated with the same volume of saline as vehicle control for additional 8 days. Lumina II living Image 4.3 software was used to analyze the fluorescence signal intensity [22]. The ethics committee of Shanghai University of Traditional Chinese Medicine approved the procedures for animal experiments.

### 2.8. Western Blotting

After treatment with or without HDSB11 (0.5, 1 mg/ml) for 24 h, each group's total protein was extracted separately and measured using a BCA protein assay kit. Protein samples (30 *μ*g) were subjected to SDS-PAGE gel electrophoresis. After 2 h of membrane transfer, PVDF membranes were blocked with 5% skim milk for 1 h, and then, the primary antibody was incubated at 4°C overnight. The nonspecific bindings of the primary antibody were washed off with TBST buffer, and membranes were incubated with the secondary antibody at room temperature for 1 h. Nonspecifically bound secondary antibodies were also eluted using TBST buffer. Protein bands were detected using the Tanon imaging system (Tanon, Shanghai, China), and the band density was semiquantitatively analyzed using the Tanon program.

### 2.9. Statistical Analysis

Statistical analysis was performed using SPSS 20.0 software (Chicago, USA). Each result is expressed as mean ± standard deviation (SD), and experiments were repeated at least three times independently. A single-factor analysis of variance between groups was analyzed, and *P* < 0.05 was considered statistically significant.

## 3. Results

### 3.1. HDSB11 Showed the Strongest Inhibition on Proliferation of LLC Cells among Extracts without Cytotoxicity on Normal Cells

**Figure 1:**
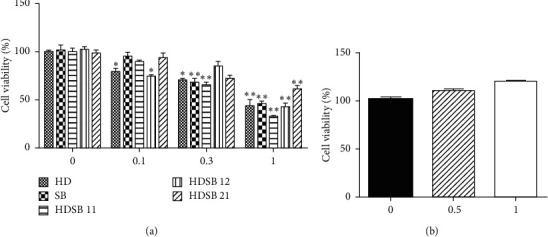
Effect of extracts from HDSB on the proliferation of LLC cells and normal lung epithelial cells. The MTT assay was carried out on LLC or BEAS-2B cells treated with different extracts or HDSB11. Values are the mean ± SD of three replicate experiments. Compared with the control group, ^*∗*^*P* < 0.05 and ^*∗∗*^*P* < 0.01.

Then, MTT assay was used to compare the toxicity effect of different extracts from HD and SB on LLC cells. The IC_50_ of HD, SB, HDSB11, HDSB12, and HDSB21 was 0.82, 0.77, 0.43, 1.08, and 1.69 mg/ml after treatment with mentioned extracts for 72 h, respectively. Among them, HDSB11 showed the lowest IC_50_ on the proliferation of LLC cells (Figure 1(a)). In addition, HDSB11 showed no toxic effect on human normal lung epithelial cell line BEAS-2B at the dosage of 0.5 and 1 mg/ml (Figure 1(b)).

### 3.2. HDSB11 Concentration-Dependently Inhibited Colony Formation and Induced Apoptosis

**Figure 2:**
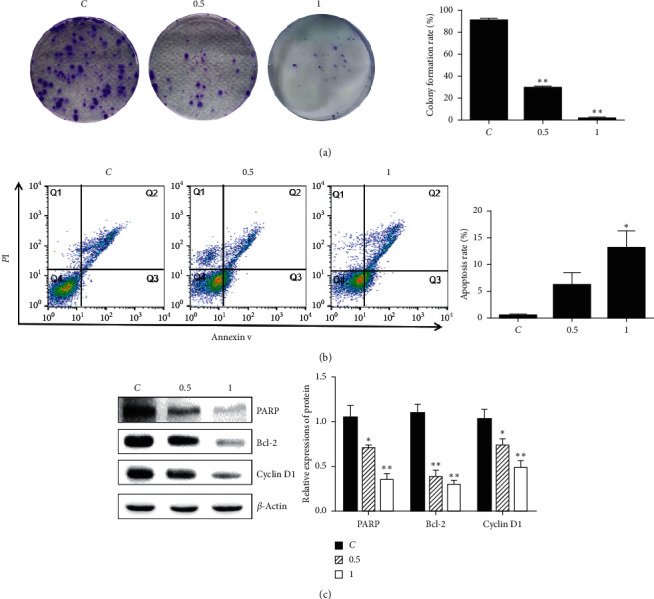
HDSB11 inhibited colony formation and induced apoptosis. After treating cells with 0.5 or 1 mg/ml HDSB11, (a) colony formation assay, (b) apoptosis assay, and (c) western blot were performed. Compared with the control group, ^*∗*^*P* < 0.05 and ^*∗∗*^*P* < 0.01.

The colony formation rate was significantly reduced after the treatment of HDSB11 (0.5 and 1 mg/ml) compared with the control group (Figure 2(a)). Flow cytometry was used to investigate whether HDSB11 induced apoptosis. The results showed that apoptotic cells increased upon HDSB11 treatment in a concentration-depended manner (Figure 2(b)).

PARP, Bcl-2, and cyclin D1 present essential functions in the apoptosis and cell cycle and have always been observed with high expressions in various cancers [23]. HDSB11 could reduce the protein expressions of PARP, Bcl-2, and cyclin D1 (Figure 2(c)).

### 3.3. HDSB11 Inhibited Tumor Growth with Decreased Expressions of NLRP3 and Cyclin D1

**Figure 3:**
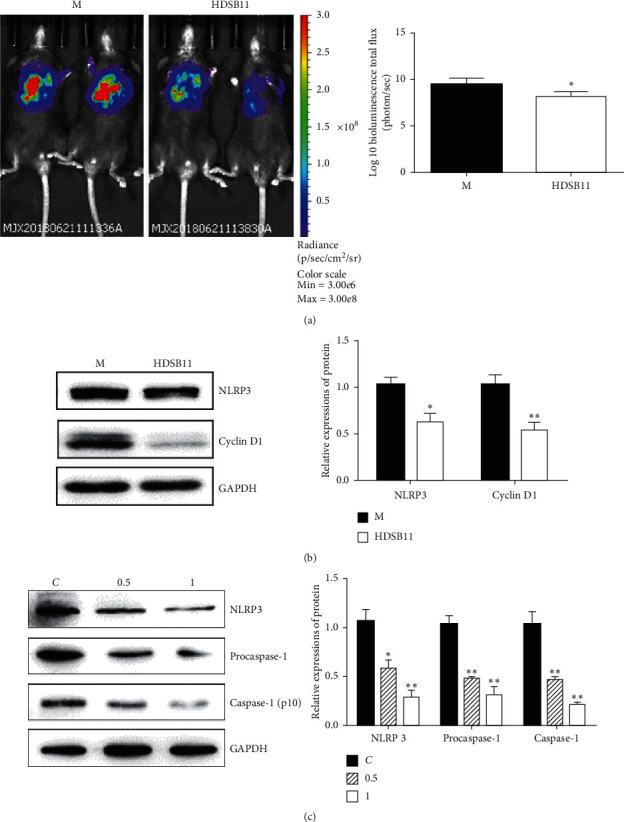
Effect of HDSB11 on tumor growth *in vivo* and expressions of NLRP3 in tumor tissues and cells. (a) *In vivo* imaging map and value; (b) NLRP3 and cyclin D1 protein expressions in tumor tissues. (c) Expressions of NLRP3, procaspase-1, and caspase-1 (p10) protein in cells. Compared with the M or C group, ^*∗*^*P* < 0.05 and ^*∗∗*^*P* < 0.01.

Compared with the model group (treated with vehicle control), the bioluminescence signals were significantly weakened in the HDSB11 group administered with 30 g crude drug/kg/d. HDSB11 could inhibit tumor growth in orthotopic lung-tumor-bearing C57BL/6 mice (Figure 3(a)).

Inhibition of NLRP3 can inactivate caspase-1 and inhibit tumor cell proliferation and migration in many kinds of cancers [9, 10, 24]. To further study the mechanism of HDSB11 in inhibition of cell proliferation and tumor growth, we tested the expression of NLRP3 in tumor tissues and cells. Western blot data showed that the HDSB11 administration inhibited NLRP3 and cyclin D1 protein expressions compared with the model group in tumor tissues (Figure 3(b)) and suppressed NLRP3, procaspase-1, and caspase-1 (p10) expressions compared with the control group in cells (Figure 3(c)).

### 3.4. HDSB11 Inactivated NF-*κ*B and MAPK Signaling Pathways

**Figure 4:**
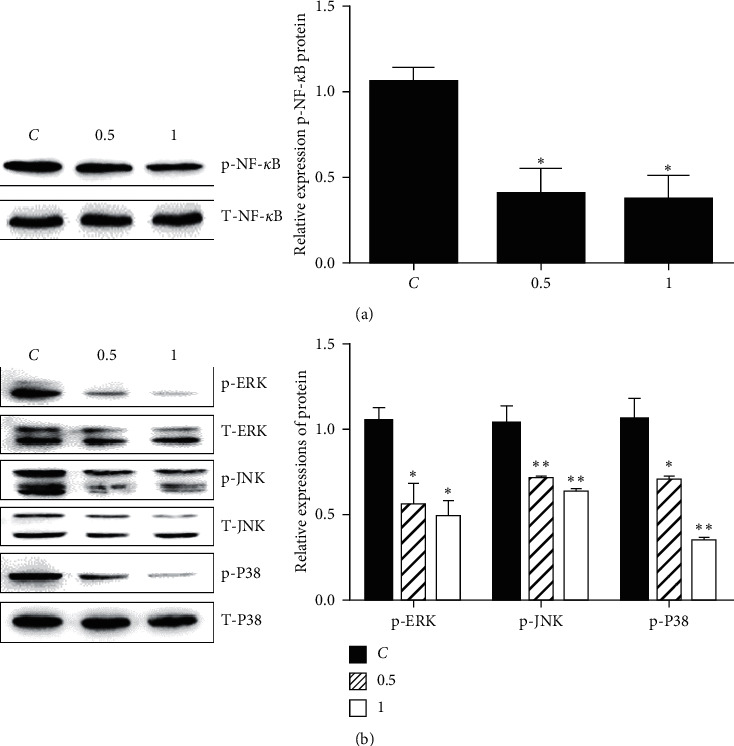
Effects of HDSB11 on NF-*κ*B and MAPK pathways. The major proteins of NF-*κ*B (a) and MAPK (b) pathways were measured by western blot after treated with HDSB11 (0.5, 1 mg/ml) for 30 min. Compared with the C group, ^*∗*^*P* < 0.05 and ^*∗∗*^*P* < 0.01.

The activation status of the NF-*κ*B signaling pathway is related to the degree of malignancy in tumors. The NF-*κ*B signaling pathway regulates NLRP3 and caspase-1 in the proliferation and migration of various cancer cells [7, 25]. HDSB11 inhibited the phosphorylated levels of NF-*κ*B, while the total NF-*κ*B level was not affected (Figure 4(a)).

Constitutively, the activated MAPK signaling pathway has been found in numerous tumors and regulates cell proliferation and apoptosis processes. The MAPK signaling pathway regulates inflammatory factors (NLRP3, ASC, caspase-1, and IL-1*β*) during the inflammatory response [26]. Data showed that HDSB11 inhibited expressions of p-ERK, p-JNK, and p-P38 MAPK without affecting the total proteins of ERK, JNK, and P38 (Figure 4(b)).

## 4. Discussion

HD and SB were combined in different weight ratios during anti-inflammatory and antitumor clinical practice in China [27].

In this study, extracts from different combinations of HD/SB were screened on LLC cells' proliferation by MTT assay. The final result showed that HDSB11 had the most significant LLC cell proliferation inhibition among these extracts and had no effect on the cell viability of normal lung epithelial cells (Figure 1). Besides, it inhibited colony formation and induced apoptosis in a concentration-depended manner (Figure 2). HDSB11 effectively reduced tumor growth on the orthotopic lung-tumor-bearing mice model (Figure 3(a)). Next, we explored the possible mechanisms underlying HDSB11-led effects.

Chronic inflammation is essential for tumor progression. Activation of NLRP3 and inflammatory cytokines promotes tumorigenesis, migration, and invasion of various tumors [28–31]. Current reports revealed that activation of NLRP3 in non-small-cell lung cancer could enhance tumor cell proliferation and migration, and NLRP3 expression inhibition could alleviate proliferation and metastasis [7]. Silencing NLPR3 inhibited tumor growth in a nude mouse endometrial tumor model, cell proliferation, migration, and invasion and reduced expression of IL-1*β* and caspase-1, while overexpression of NLPR3 led to the opposite results in endometrial cancer [10].

Experimental data demonstrated that natural products affected tumor growth and metastasis, partly regulating NLRP3 and relevant inflammatory signaling pathways. Silybin inhibited the migration of MDA-MB-231 cells by reducing the expressions of NLRP3, caspase-1, and IL-*β* proteins [32]. Berberine inhibited cell proliferation and migration of MDA-MB-231 cells via downregulating the expression of NLRP3, procaspase-1, apoptosis-related proteins, and the secretions of IL-1*α*, IL-1*β*, IL-6, and TNF-*α*. Corylin, a compound extracted from *Psoralea*, can attenuate the inflammatory response of LPS-induced BV2 cells by inhibiting the activation of the MAPK signaling pathway and reducing the expression of inflammatory factors including NLRP3, ASC, caspase-1, and IL-1*β* [26]. The expressions of NLRP3 in tumor tissues and LLC cells were significantly reduced by HDSB11 (Figure 3(b) and 3(c)).

NF-*κ*B and MAPK pathways have been reported to regulate NLRP3. Resveratrol inhibited NSCLC cells' (A549 and H1299) proliferation and migration by reduced expressions of NLRP3, ASC, and caspase-1, partly via downregulating the phosphorylated level of NF-*κ*B p65. This inhibitory effect reversed with the forced activation of NLRP3 [7]. NLRP3 and NF-*κ*B p65 were observed to have elevated expressions in malignant glioma tissues. Inhibition of NLRP3 and NF-*κ*B p65 could inhibit the growth and invasion of glioma cells. In contrast, NLRP3 overexpression promotes the growth and invasion of gliomas through IL-1*β*/NF-*κ*B p65 signaling [25]. Studies showed that inhibition of NLRP3 could reduce the proliferation and migration of A549 cells induced by LPS/ATP and reduce the protein expression of phosphorylated p38 MAPK [33]. ERK and JNK are overactivated in many tumors. Inhibition of continued activation of ERK and JNK could inhibit cell proliferation in non-small-cell lung cancer cells [34, 35]. In this study, HDSB11 could simultaneously inactivate NF-*κ*B and MAPK signaling pathways (Figure 4).

In conclusion, HDSB11 could inhibit tumor growth, cell proliferation, and colony formation and induce apoptosis on cells and the animal model. The antitumor effect was achieved by inhibiting the expression of NLRP3 and related inflammatory factors and inactivation of the NF-*κ*B/MAPK signaling pathways.

## Data Availability

The data used to support the findings of this study are included in the article.
